# Terminal Glaucoma Mimicking Posterior Cerebral Artery Infarction: A Case Report

**DOI:** 10.7759/cureus.88358

**Published:** 2025-07-20

**Authors:** Shin-Rung Tsai, Wei-Hao Lin

**Affiliations:** 1 Neurology, Kaohsiung Medical University Hospital, Kaohsiung, TWN

**Keywords:** case reports, charles-bonnet syndrome, glaucoma, hallucinations, hemianopsia

## Abstract

Visual disturbances such as homonymous hemianopia and visual hallucinations often suggest central nervous system pathology, particularly occipital lobe infarction. However, ocular conditions may present similarly and lead to misdiagnosis. We report the case of a 73-year-old man presenting with right-sided visual field deficits and visual hallucinations, initially suggestive of posterior cerebral artery stroke. Neuroimaging and electroencephalograms were unremarkable. Further ophthalmologic evaluation revealed advanced normal-tension glaucoma (NTG) with bilateral field constriction and significant optic nerve cupping, consistent with terminal-stage NTG. The visual hallucinations were attributed to Charles Bonnet syndrome. This case underscores the importance of including ocular etiologies in the differential diagnosis of stroke mimics, particularly in elderly patients with visual complaints.

## Introduction

Visual field deficits and hallucinations are often indicative of central nervous system (CNS) lesions, particularly those affecting the occipital lobe or optic radiation. While stroke is the leading cause of homonymous hemianopia (HH) in adults [1], ophthalmologic conditions such as glaucoma may also produce visual deficits and mimic neurological disease. Charles Bonnet syndrome (CBS), characterized by visual hallucinations (VH) in patients with significant visual loss due to diseases affecting the visual pathway, can further complicate the clinical picture. These hallucinations may be simple (e.g., shapes, flashes of light) or complex (e.g., people, animals, or scenes) [2]. This case illustrates how terminal-stage normal-tension glaucoma (NTG) can mimic a posterior cerebral artery (PCA) infarction and presents a diagnostic challenge that requires interdisciplinary evaluation. This article was previously presented as a poster at the 5th International Taiwanese Congress of Neurology & Annual Meetings of the Taiwan Neurological Society on April 25, 2025.

## Case presentation

A 73-year-old man presented with a sudden onset of visual disturbances, describing colorful bubbles and silhouettes in the right visual field, affecting both eyes. He also experienced spatial disorientation, often reaching inaccurately to his right side. He denied headache, fever, or other neurological symptoms. His medical history included ischemic heart disease with coronary stenting 15 years prior, poorly controlled diabetes mellitus, and hyperlipidemia. He was not on any regular medications.

His vital signs were stable. Neurological examination revealed right HH without other focal deficits. A left occipital lesion was initially suspected, with differential diagnoses including cerebral infarction, neoplasm, or seizure. Laboratory tests showed hyperglycemia and impaired renal function. Brain magnetic resonance imaging revealed no acute abnormalities, and the electroencephalogram (EEG) was unremarkable.

**Figure 1:**
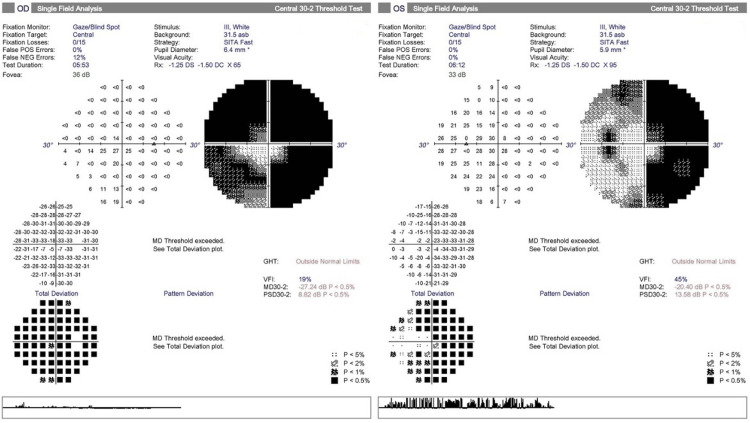
Automated perimetry revealed tunnel vision in the right eye and an arcuate visual field defect in the left eye.

**Figure 2:**
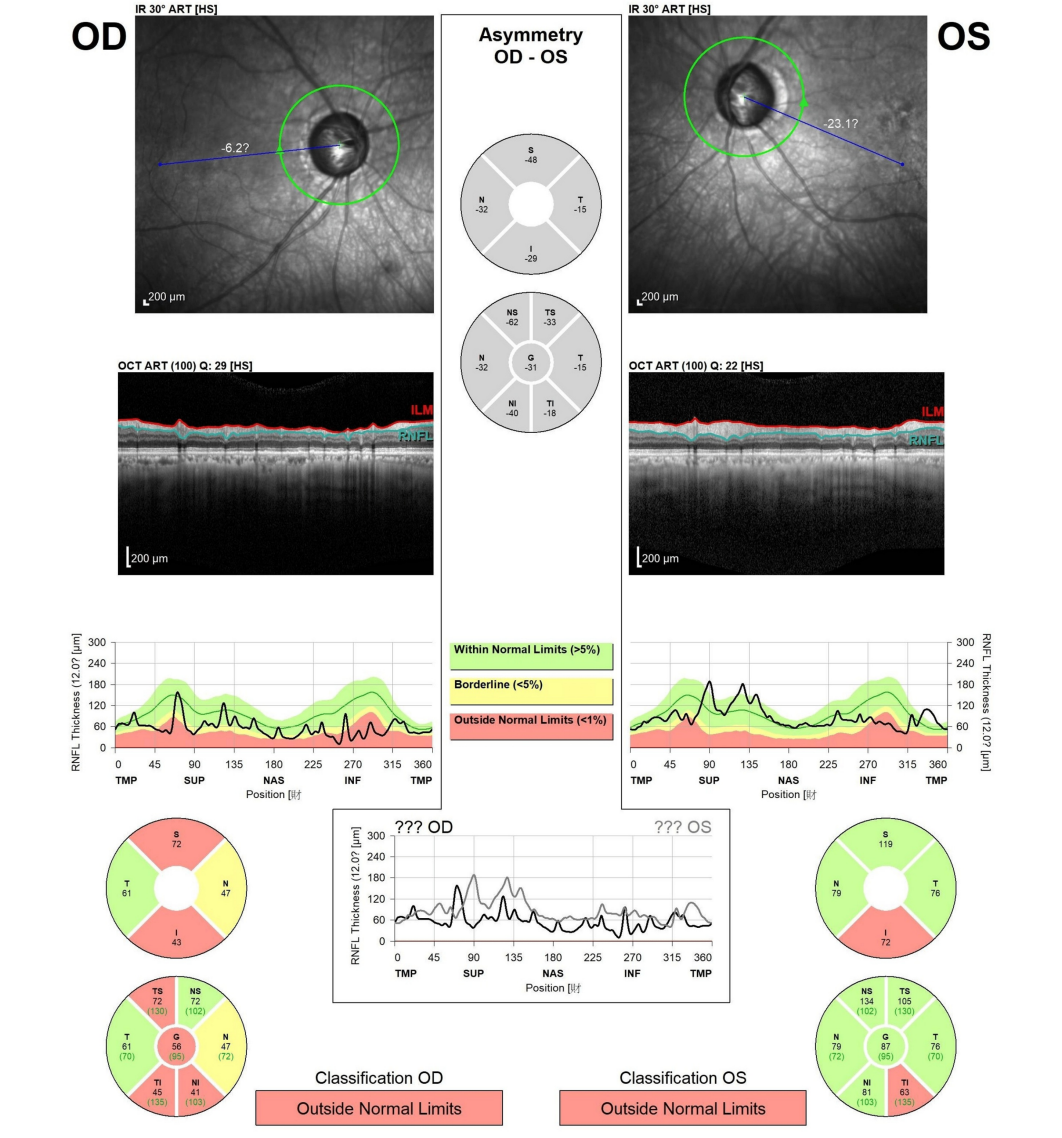
Optical coherence tomography (OCT) revealed diffuse retinal nerve fiber layer thinning in the right eye with relative preservation in the temporal region. The left eye demonstrated mild thinning in the superotemporal region and moderate to severe thinning in the inferotemporal region.

Ophthalmologic evaluation revealed intraocular pressures (IOP) of 17.2 mmHg (right eye) and 14.9 mmHg (left eye), with bilateral optic nerve cupping (cup-to-disc ratio of 0.8). Automated perimetry showed tunnel vision in the right eye and an arcuate defect in the left (Figure 1). Optical coherence tomography confirmed retinal nerve fiber layer thinning (Figure 2), and visual evoked potentials suggested prechiasmatic pathology. These findings supported a diagnosis of advanced normal-tension glaucoma (NTG) with binocular visual field loss mimicking HH. The VH was consistent with CBS.

Treatment included brimonidine tartrate eye drops for IOP reduction and clonazepam 0.5 mg daily for CBS. The patient reported resolution of hallucinations and some improvement in spatial orientation. He was discharged in stable condition with ophthalmologic follow-up.

## Discussion

HH is defined as a visual field defect affecting the same side of both eyes, typically resulting from lesions in the visual pathway posterior to the optic chiasm. The most commonly affected sites are the occipital lobe (45%) and optic radiations (32%), with stroke being the predominant cause in adults (52-70%), particularly PCA infarctions. Other etiologies include traumatic brain injury (14%) and tumors (11%) [1]. HH caused by ophthalmic conditions is exceedingly rare. In this case, the patient’s visual field defects and hallucinations initially raised concern for PCA infarction but were ultimately attributed to advanced NTG and CBS. The patient exhibited tunnel vision in the right eye and an arcuate scotoma in the left eye, typical patterns of visual field loss in glaucoma, characterized by peripheral vision impairment [3]. The coincidental alignment of these defects may have mimicked homonymous hemianopia during the initial neurological evaluation.

The presence of VH can further complicate lesion localization. A previous study suggested that VH may arise from phasic increases in activity within specialized regions of the visual cortex, each corresponding to specific visual functions. For instance, object-related hallucinations involve the object-processing cortex. Additionally, the characteristics of VH often align with specific visual pathways: the ventral temporal pathway for scenes and figures, the superior temporal sulcus for facial imagery, and the dorsal parietal pathway for phenomena such as visual perseveration and palinopsia [4]. Beyond CNS etiologies, visual impairment from ophthalmic diseases can also lead to VH through a deafferentation-hyperexcitability mechanism, which is considered the underlying pathophysiology of CBS [2,5].

CBS is a type of release hallucination, meaning that hallucinations arise as the brain attempts to compensate for the absence of visual input, which is consistent with the deafferentation hyperexcitability model [5,6]. It is primarily caused by acquired visual impairment and can originate from any lesion along the visual pathway, from the visual cortex to the eyes. Although it can affect individuals of all ages, it is more commonly seen in older adults who are more likely to experience significant vision loss due to ocular diseases or occipital stroke. The hallmark features of CBS include simple or complex visual hallucinations that are usually more noticeable when the eyes are open, preserved insight, and the absence of other sensory hallucinations. Patients with CBS typically have no cognitive impairment, psychiatric illness, or other neurological disorders [6]. Among patients with glaucoma, the prevalence of CBS is approximately 2.8%, increasing to 20.1% in those with severe visual impairment [7]. 

While most cases of CBS are associated with severe visual impairment, identifiable triggering or relieving factors are uncommon [8]. However, not all patients with low vision develop CBS. Shiraishi et al. suggested that a dynamic or acute reduction in visual acuity may play a more significant role in the onset of CBS than static, chronic visual impairment [9]. In our patient with advanced glaucoma, the gradual peripheral field constriction may have gone unnoticed, allowing for neural adaptation. Nevertheless, once visual input declined beyond a critical threshold, visual hallucinations emerged. However, this remains speculative and warrants further investigation.

NTG, a subtype of open-angle glaucoma, is characterized by optic nerve damage and progressive visual field loss despite IOP remaining within the normal range. Its multifactorial pathogenesis includes aging, genetic factors, and vascular dysregulation [10]. NTG is more prevalent in Asian than Western populations [11]. Clinical features of NTG include large optic disc size, optic nerve cupping, peripapillary pigment changes, optic disc hemorrhages, retinal nerve fiber layer loss, and visual field defects. Although IOP is normal, lowering IOP remains the mainstay of treatment, as a reduction of at least 30% has been shown to slow disease progression [12]. Many NTG patients retain good central visual acuity despite significant peripheral field loss [13], which likely contributed to the underrecognition of visual disability in our patient. The observed spatial disorientation was likely due to peripheral field constriction, a typical manifestation of advanced glaucoma [14].

Management of CBS focuses on addressing the underlying visual impairment and providing symptomatic relief from hallucinations. In this case, IOP was managed with brimonidine tartrate eye drops, while clonazepam was prescribed to alleviate VH. The subsequent improvement in spatial orientation and resolution of hallucinations supported the diagnosis of NTG-associated CBS rather than a CNS pathology. Although the initial clinical picture was suggestive of a PCA stroke, the exclusion of central causes led to the recognition of advanced NTG. This case underscores the importance of considering ocular causes when evaluating patients with visual field defects and VH, particularly when CNS pathology has been ruled out. To the best of our knowledge, this is the first reported case of bilateral glaucoma presenting with visual field defects that coincidentally resembled homonymous hemianopia.

## Conclusions

This case presents a rare instance of NTG with CBS mimicking a PCA infarction, highlighting the clinical challenge of distinguishing ophthalmologic from neurologic etiologies in patients presenting with visual field deficits and VH. Despite stroke-like symptoms and HH suggestive of CNS involvement, thorough neuroimaging failed to reveal acute pathology. Subsequent ophthalmologic evaluation uncovered advanced NTG as the true cause of the visual disturbance, with CBS accounting for the hallucinations. This case underscores the importance of considering ocular causes, especially advanced glaucoma, in the differential diagnosis when central causes are excluded. Comprehensive ophthalmologic assessment and timely diagnosis are crucial for initiating appropriate treatment and avoiding misdiagnosis.
